# Senescence of song revealed by a long-term study of the Seychelles warbler (*Acrocephalus sechellensis*)

**DOI:** 10.1038/s41598-020-77405-3

**Published:** 2020-11-24

**Authors:** Mathew L. Berg, Sarah C. Beebe, Jan Komdeur, Adam P. A. Cardilini, Raoul F. H. Ribot, Andrew T. D. Bennett, Katherine L. Buchanan

**Affiliations:** 1 Centre for Integrative Ecology, School of Life and Environmental Sciences, Deakin University, Locked Bag 20000, Geelong, VIC 3220 Australia; 2 Groningen Institute for Evolutionary Life Sciences, Faculty of Science and Engineering, University of Groningen, Nijenborgh 7, 9747 AG Groningen, The Netherlands; 3 Department of Zoology, University of Cambridge, Downing Street, Cambridge, CB2 4EJ UK; 4 Nature Seychelles, Victoria, Mahé, Seychelles

**Keywords:** Behavioural ecology, Sexual selection

## Abstract

Senescence is widespread in nature, often resulting in diminishing survival or reproduction with age, but its role in age-dependent variation in sexual traits is often poorly understood. One reason is that few studies of sexual traits consider non-linear relationships with age, or only consider a narrow range of years relative to the life span of the species. Birdsong has evolved to allow assessment of conspecific quality in numerous bird species. Whilst theory and empirical work suggests that song may become more elaborate with age, there are a paucity of long-term studies testing whether song is associated with age or longevity. In particular, the occurrence of song senescence has rarely been demonstrated. Using an exceptional long-term dataset for the Seychelles warbler (*Acrocephalus sechellensis*), we analysed relationships between male song, age, survival, and longevity. This species is a long-lived songbird with early life increases, followed by senescent declines, in survival and reproduction. The study population (Cousin Island, Seychelles) is a closed population, with no depredation of adults, providing an excellent opportunity to study senescence in free-living animals. We tested whether song traits were related to age at recording, future survival, longevity, and territory quality. We found age-dependent changes in five song traits (duration, maximum frequency, peak frequency of songs, and duration and frequency bandwidth of trills). Relationships with age were quadratic, indicating reversal in the expression of song coinciding with the onset of senescence in reproduction and survival in this species. One song trait (trill bandwidth) had a quadratic relationship with future survival, but no song traits were related to longevity, suggesting age-related patterns were not the result of selective disappearance. Our study provides one of the first examples of functional senescence in song, offering new insights into avian senescence. Late-life declines in avian song, and possibly other sexual traits, may be more common than currently known, and may play a fundamental role in age-dependent changes in reproductive success.

## Introduction

Vocal communication plays an integral role in mate attraction, male-male competition, territory defence, and identification of conspecifics^1–4^. Evolutionary theory predicts that sexual selection should favour more elaborate traits which are costly to produce and maintain, so providing honest indicators of the signaller’s quality^5^. Age-related changes in trait quality can reinforce the honesty of signals, and can highlight mechanisms underlying trait production^6,7^. There is evidence that traits such as repertoire size, song consistency, trill rate, and vocal deviation (the trade-off between trill rate and frequency bandwidth) are age-dependent^7–10^. Age-related changes in vocal production may also be indicative of an individual’s physical condition and longevity^7,10,11^. For example, Reid et al.^12^ demonstrated that male song sparrows (*Melospiza melodia*) with larger song repertoires were longer lived and sired more independent and recruited offspring and grand-offspring. Forstmeier et al.^10^ showed that syllable switching was positively associated with life span in great reed warblers (*Acrocephalus arundinaceus*), and in male sedge warblers (*A. schoenobaenus*), Nicholson et al.^11^ found that repertoire size increased from year to year in individual males, although there was no relationship between male age and repertoire size overall.

Senescence (deterioration in old age) in physiological functions is thought to occur in most species and often results in age-dependent declines in survival or reproduction^13,14^, but little is known about senescent patterns in sexual signals such as song^15–19^. For song, most studies examining age-related changes have focussed on determining whether traits change in a linear manner, usually over a narrow range of years relative to the life span of the species^10,11^. However, it is possible that vocal traits may show non-linear relationships with age, due to senescence in the physical, motor or neural ability to produce songs^8,20^. Moreover, observations of senescence are most likely in captive populations or from long-term studies of natural populations of long-lived species with high survival, because high extrinsic mortality is often sufficient to obscure observations of senescent patterns, and this has limited our understanding of the evolutionary and ecological consequences of senescence^15,21^. There are two potential approaches for examining associations between age and song traits, which may reveal senescent patterns. First, a longitudinal approach may be adopted which looks for temporal changes in song traits during the lifetime of known individuals^12^. The second possibility is a cross-sectional approach, where song characteristics are analysed for different individuals of known ages, at a single point in time^20,22,23^. With the second approach, individuals with certain traits may have reduced viability and therefore be underrepresented in old age classes due to selective mortality rather than functional senescence^12^. However, any selective (dis)appearance may be revealed by relating traits of interest to longevity (survival) if such data are available^24,25^. Years until death may also be modelled to test for linear or accelerating declines independent of age^25^.

To our knowledge, possible senescence in bird song has only been documented in four species previously^8,20,26–28^. Three of these species were studied using songs recorded in captive populations, and documented late life deterioration of (i) repertoire size in female European starlings (*Sturnus vulgaris*)^26^, (ii) tempo and frequency parameters of song in male Bengalese finches (*Lonchura striata*)^20^, and (iii) song rate, stereotypy within songs, song consistency, and response elicited by song playback in swamp sparrows (*Melospiza georgiana*)^27,28^. Late-life declines in song of wild birds has been reported by Rivera-Gutierrez et al.^8^, who used a longitudinal approach to study two song variables in male great tits (*Parus major*). Rivera-Gutierrez et al.^8^ found that while repertoire size remained constant among years, song consistency increased in younger birds but decreased in older birds.

The Seychelles warbler (*A. sechellensis*), an insectivorous songbird endemic to the Seychelles islands^29^, is an excellent model for studying age-dependence. It comprises closed island populations^30^ where nearly all individuals are marked and have been monitored at least annually from birth to death, from 1985 to the present^31^. The species shows virtually no inter-island dispersal^30^ and an absence of adult predation, resulting in little extrinsic mortality and 84% annual adult survival^13,14,32^. Seychelles warblers are unusually long-lived for a small passerine, with an average lifespan of around 5 years, and some have been recorded to survive 17 years^33,34^. Once paired, warblers usually remain on the same breeding territory throughout their life^29,35^. The Seychelles warbler is a facultatively cooperative breeder, with some territories containing a breeding pair as well as subordinate helpers that are also sexually mature birds^13,29,35^. Only one previous study has documented the acoustic structure of the song of the Seychelles warbler, showing that the species has an unusually narrow frequency bandwidth for an *Acrocephalus* species and an unusually large repertoire size^36^. The unique long-term monitoring and closed island populations of the Seychelles warbler mean that unusually powerful tests of how song changes with age, or whether song traits can predict survival or longevity, are possible.

In this study, we used historical song recordings^36^ from the Cousin population of Seychelles warblers to test for both linear and quadratic relationships between male song traits and (i) age of each male at time of recording, (ii) years until death of each male at time recording, and (iii) longevity (life span) of each male. Survival and reproductive senescence have been previously documented in Seychelles warblers; early life increases in reproductive output (fledgling production) lead to a peak at about 6 years of age in females and between 6 and 9 years in males, followed by a terminal decline^13,14,31,37^. Because male song is important for mate choice and male fitness in *Acrocephalus* species^38,39^, we hypothesised that acoustic variation of song might follow a parallel quadratic age-related pattern. In addition, we tested the influence of territory quality (size and food availability) on song traits. We hypothesised that territory quality would be related to acoustic parameters of song, such that high quality males would both hold high quality territories and produce more elaborate song traits.

## Results

### Age, years before death and longevity

**Figure 1:**
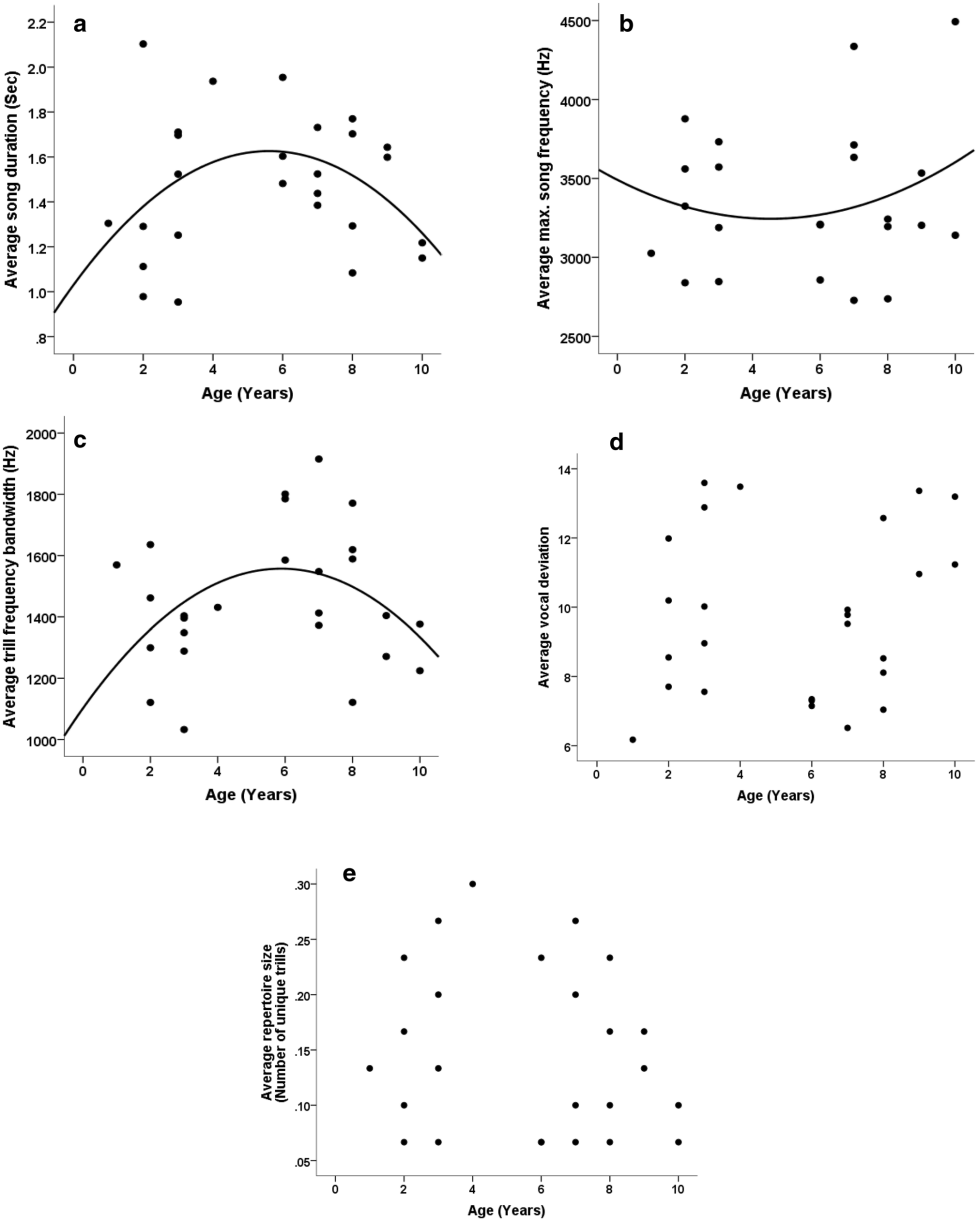
Age-related changes in song parameters in the Cousin Island population of Seychelles warblers. Black dots represent the mean value for an individual territory owning male (n = 31). Quadratic regression lines are depicted for illustrative purposes, and indicate significant quadratic relationships found using general linear models (see Table 1). (**a**,**b**) Show the onset of senescent deteriorations on song traits, which peak around 6 years, and (**c**) which peaks around 5 years. (**d**,**e**) No relationship between age and either vocal deviation or repertoire size was found (see Table 1).

The mean age of Seychelles warbler males at the time of recording was 5.54 ± 2.85 years (range 1–10 years, n = 35). Age followed a significant quadratic relationship in five vocal traits, which included song duration, trill duration, peak song frequency, maximum song frequency, and trill bandwidth (Table 1, Fig. 1). The probability of finding a significant result for five or more out of 15 independent song traits by chance alone is 0.00061, so taken together the results strongly supported a quadratic relationship between age and song. Four of these song traits had a negative quadratic relationship with age, increasing in younger males, peaking around 6 years of age, and decreasing with age in older males. The exception was maximum song frequency, which showed a positive quadratic relationship where maximum frequency decreased with age until around 5 years of age, before increasing with age in older males (Fig. 1b). Marginally non-significant quadratic trends were also observed between male age and song frequency bandwidth, and peak trill frequency (Table 1), where values for these vocal traits increased in young males, peaked around 6 years, and decreased in late-life. We found no age-related changes in vocal deviation of trills, minimum vocal deviation, mean male vocal deviation (Fig. 1d), or repertoire size (Fig. 1e).

**Table 1:** Associations of age (showing linear and quadratic contrasts), territory quality, and body mass with song traits in male Seychelles warblers.

Song trait	Predictors	Estimate	SE	*t*	df	*P*
**Trill duration (s)**	Age (linear)	− 4.93	2.23	− 2.21	3.42	0.103
	**Age (quadratic)**	− 7.55	2.56	− 2.95	7.34	**0.020**
	Territory quality	− 0.03	0.03	− 0.86	2.83	0.459
	Mass	− 0.19	0.07	− 2.84	3.50	0.055
**Song duration (s)**	Age (linear)	− 10.18	3.27	− 3.11	2.99	0.053
	**Age (quadratic)**	− 13.07	3.74	− 3.49	6.52	**0.011**
	Territory quality	0.02	0.05	0.34	2.93	0.756
	**Mass**	− 0.39	0.10	− 3.90	3.12	**0.028**
Trill rate (Hz)	Age (linear)	− 11.85	24.00	− 0.49	1.83	0.674
	Age (quadratic)	25.93	27.48	0.94	3.96	0.399
	Territory quality	1.10	0.36	3.02	1.52	0.130
	Mass	0.80	0.72	1.10	1.88	0.393
Min. song frequency (Hz)	Age (linear)	33.01	1111.61	0.03	4.18	0.978
	Age (quadratic)	− 926.88	0.03	− 0.69	6.34	0.514
	Territory quality	24.33	18.60	1.31	4.87	0.249
	Mass	10.06	37.77	0.27	3.93	0.803
**Max. song frequency (Hz)**	**Age (linear)**	− 4536.48	2266.29	− 2.00	80.00	**0.049**
	**Age (quadratic)**	− 8273.96	3107.61	− 2.66	80.00	**0.009**
	**Territory quality**	100.64	39.11	2.57	80.00	**0.012**
	**Mass**	− 223.98	75.33	− 2.87	80.00	**0.004**
Song bandwidth (Hz)	Age (linear)	− 3783.17	2724.26	− 1.39	3.32	0.251
	Age (quadratic)	− 7068.12	2957.47	− 2.39	5.87	0.055
	Territory quality	77.85	42.80	1.82	3.48	0.154
	Mass	− 165.89	82.19	− 2.02	3.52	0.123
Min. trill frequency (Hz)	Age (linear)	229.16	3245.91	0.07	5.78	0.946
	Age (quadratic)	1024.88	3300.16	0.31	7.83	0.764
	Territory quality	42.80	49.94	0.88	5.82	0.415
	Mass	103.94	96.87	1.07	6.04	0.324
Max. trill frequency (Hz)	Age (linear)	− 3973.05	4917.64	− 0.81	6.51	0.448
	Age (quadratic)	− 730.18	4908.22	− 0.15	5.07	0.887
	Territory quality	28.70	75.48	0.38	5.13	0.719
	Mass	41.75	146.15	0.29	5.27	0.786
**Trill bandwidth (Hz)**	Age (linear)	− 2410.83	2117.60	− 1.14	2.70	0.346
	**Age (quadratic)**	− 6919.69	2520.26	− 2.75	6.72	**0.030**
	Territory quality	7.50	31.66	0.24	1.96	0.835
	Mass	− 130.14	63.97	− 2.03	2.69	0.145
Peak trill frequency (Hz)	Age (linear)	− 1417.67	3340.24	− 0.42	5.05	0.689
	Age (quadratic)	− 7646.32	3400.26	− 2.25	6.88	0.060
	Territory quality	59.76	51.40	1.16	5.08	0.297
	Mass	− 133.38	99.70	− 1.34	5.27	0.236
**Peak song frequency (Hz)**	Age (linear)	− 3284.62	2821.42	− 1.16	4.15	0.307
	**Age (quadratic)**	− 7806.64	3028.54	− 2.58	6.98	**0.037**
	Territory quality	28.83	44.27	0.65	4.38	0.547
	Mass	− 141.36	85.01	− 1.66	4.40	0.165
Vocal deviation	Age (linear)	25.93	25.07	1.03	3.98	0.360
	Age (quadratic)	55.96	30.02	1.86	9.96	0.092
	Territory quality	− 0.77	0.37	− 2.07	2.82	0.136
	Mass	0.83	0.76	1.10	3.94	0.335
Minimum vocal deviation	Age (linear)	37.13	33.80	1.10	6.00	0.314
	Age (quadratic)	57.96	31.75	1.83	6.00	0.118
	Territory quality	− 0.59	0.52	− 1.15	6.00	0.295
	Mass	− 0.12	0.99	− 0.12	6.00	0.908
Mean vocal deviation	Age (linear)	14.42	29.47	0.49	6.00	0.642
	Age (quadratic)	49.74	27.68	1.80	6.00	0.122
	Territory quality	− 0.80	0.45	− 1.78	6.00	0.125
	Mass	0.30	0.87	0.35	6.00	0.738
Repertoire size	Age (linear)	− 1.22	1.29	− 0.95	6.00	0.381
	Age (quadratic)	− 1.13	1.22	− 0.93	6.00	0.387
	Territory quality	0.02	0.02	0.77	6.00	0.473
	Mass	− 0.02	0.04	− 0.41	6.00	0.698

Significant predictors (P < 0.05) are highlighted in bold.

The mean YBD (years before death) and longevity of males included in our analyses were 4.50 ± 4.08 years (range 0–14 years) and 10.04 ± 3.75 years (range 2–16 years), respectively. As with age, YBD had a negative quadratic association with trill bandwidth, but no linear or quadratic relationships with any other vocal traits (see Supplementary Table S1 online). The probability of finding one or more significant results out of 15 tests by chance alone is 0.54, so these results do not strongly support an association between song and YBD. Longevity was unrelated linearly or quadratically to any vocal trait (see Supplementary Table S2 online). The probability of finding none or one significant relationship out of 15 tests by chance is 0.463 and 0.366, respectively, so overall the data did not provide strong support for the hypotheses that YBD or longevity were related to song.

### Territory quality

Territory quality had no consistent relationships with vocal traits in our models, but was significantly related to maximum song frequency when controlling for age (Table 1), trill rate and trill bandwidth when controlling for YBD (see Supplementary Table S1 online), and trill duration when controlling for longevity (see Supplementary Table S2 online). These results suggest that males in higher quality territories produced songs with a higher trill rate, shorter trill duration, narrower trill frequency bandwidth, and higher maximum frequency, than males in lower quality territories.

We repeated the analysis with territory quality separated into its two constituent components: (i) territory size, and (ii) food availability^40^, whilst controlling for age and body mass. This indicated that the food availability component had significant relationships with maximum trill frequency and trill rate (see Supplementary Table S3 online); males on territories with higher food availability produced more syllables per second and trills with a lower maximum frequency. In contrast, territory size was related to minimum and maximum song frequency, with males in larger territories producing higher minimum and maximum song frequencies compared to males in smaller territories (see Supplementary Table S3 online). Interactions of age by territory quality were non-significant in all except one case: there was a significant age by territory quality interaction on repertoire size (*P* = 0.04), where repertoire size was unrelated to territory quality in younger males but older males in high quality territories produced smaller repertoire sizes than those in low quality territories.

## Discussion

Animal vocalisations such as avian songs play well established roles in mate attraction, male-male competition, territory defence, and identification of individuals or conspecifics, but the possibility of senescence in such vocalisations, or other sexual displays, remains largely unconfirmed^8,15–19^. Our study demonstrates quadratic age-dependent variation in song in a closed population of male Seychelles warblers, and documents senescent change with a similar age of onset in the expression of several temporal- and frequency-related vocal traits (not including vocal deviation or repertoire size). Our cross-sectional analyses revealed that both temporal and frequency parameters of song varied with age in young males, reached an asymptote around 6 years of age, and reversed with age in older males. Notably, our findings on song traits are concordant with patterns of reproductive and survival senescence which have previously been reported in Seychelles warblers, and which show a peak in reproductive output followed by the onset of reproductive senescence occurring between 6 and 9 years of age in males, or between 6 and 7 years of age in females^13,14,31^.

Song performance is important in determining male fitness for other *Acrocephalus* through female choice, extra-pair paternity and territory defence, although the role of acoustic traits in mediating these fitness determinants has not been studied in Seychelles warblers^38,39^. Thus, it is possible that reproductive and survival senescence in Seychelles warblers may be mediated at least in part by parallel senescence in song, particularly in light of the intense competition for extra-group fertilisations which male Seychelles warblers are likely to experience (approximately 44% of offspring fathered by dominant males from other territories^14,41,42^), although further work is required to test this hypothesis. We found that most song variables such as duration and bandwidth increased early in life followed by later life declines. Evidence from other species suggests that greater song duration and bandwidth may characterise high quality songs, for example because they are favoured by females^4,23,43^. Interestingly, we found maximum song frequency declined in early in life followed by an increase in later life. It is therefore possible that lower maximum frequency represents higher song quality in Seychelles warblers. Alternatively, it may be that maximum frequency must be traded-off against other age-dependent song traits, or that differences in body size among age classes constrains maximum frequency^44^. Further research is required to understand the acoustic features that determine song quality and how they are related to reproductive success in Seychelles warblers.

In marked contrast to the age-related relationships, only one vocal trait (trill bandwidth) had a significant relationship (quadratic) with YBD. We found no relationships (linear or quadratic) between vocal traits and longevity, indicating that none of the song traits we measured were associated with lifetime survival and that selective disappearance is unlikely to explain the senescent patterns that we observed. These findings contrast with some previous studies, for example in song sparrows (*Melospiza melodia*)^12^ and great reed warblers^10^, where repertoire size and syllable switching, respectively, were correlated with male longevity. If any clear relationships between song traits and individual survival or longevity exist, in addition to age-dependence, there are two reasons we may expect them to be evident in our study. First, because we analysed a long-term dataset of a closed island population which provided reliable information on survival, and second, because the variation in age at recording of birds included in our study reflects the typical natural lifespan of Seychelles warblers. On the other hand, it is possible that a larger sample size of songs, or songs recorded over a longer time period than was possible for this study, may reveal relationships with longevity that are currently not apparent. Additionally, associations between song quality and survival may be more likely in populations with substantial predation of adults, unlike Seychelles warblers, if predators remove lower quality individuals with lower quality songs from the population at a higher rate than higher quality individuals. It would be interesting to compare associations between song and longevity in populations with higher predation of adult birds than Cousin Island.

It seems plausible that senescent patterns in vocal quality, or other sexual displays, are widespread among species, but poorly documented due to a paucity of long-term studies where such traits are measured in individuals that are followed from birth, and thus of known age and longevity^16,17^. All studies that have previously reported senescent patterns in avian song used longitudinal analyses to compare song traits with age^8,20,26–28^, but only one study was conducted using songs recorded from free-living birds and analysed an estimate of longevity as well^8^. Patterns we found in the Seychelles warbler, including delayed maturation followed by late-life reversal in some song traits and the limited relationship between song traits and longevity, were similar to senescent patterns in a population of wild great tits^8^, although Seychelles warbler males in our study were analysed over longer lifespans than the males analysed in the great tit study (10 years vs 6 years). In swamp sparrows, Zipple et al.^27^ reported that neither song duration or vocal deviation declined as males aged, although some other acoustic and behavioural aspects of song did^27,28^. In Bengalese finches, Cooper et al.^20^ found that as males aged their songs had narrower frequency bandwidth, which is similar to our findings in Seychelles warblers, as well as lower pitch and longer inter-syllable duration. As in the great tit study^8^, we found no evidence in Seychelles warblers for age-related changes in repertoire size, including declines in repertoire size in later life. This contrasts to some extent with studies in other *Acrocephalus* species, where age-related changes in song complexity have been reported. Forstmeier et al.^10^ found that male great reed warblers increased their repertoire size as they got older, but in only one of the two populations studied. In male sedge warblers (*A. schoenobaenus*), a longitudinal analysis found that repertoire size increased in successive years in individual males, but there was no relationship between male age and repertoire size overall, and the study was limited by a small sample size, analysis of a limited age range, and uncertainty in the exact ages of birds^11^.

The proximate explanations for senescent patterns in song traits in birds remain unknown. One hypothesis involves morphological or neural degeneration in later life leading to a deterioration in motor performance^20,45^. In our study, vocal deviation showed no relationships with age, YBD or longevity. This was surprising, as vocal deviation is a song trait that is considered to be strongly linked to motor skills because it is difficult for birds to produce trilled syllables both rapidly and with a wide frequency bandwidth^46^. Although taxonomically distant, songbirds and humans share many commonalities in the control of their vocal learning, meaning that bird song represents a valuable model for understanding speech and language control in humans^20,45,47^. Our findings reveal some interesting parallels in age-related changes in vocalisation. In both Seychelles warbler males and aging human males^48^, maximum frequency of an individual’s acoustic signal increases in young males, and then declines in older individuals. However, while fundamental frequency increases in aging male humans^49^, possibly due to changes in testosterone^50,51^, it declines in older male Seychelles warblers. A possible mechanism underlying these late-life changes in humans is thought to be changes in lung function^48^. Whereas little is known about age-related changes in either lung or syringeal function in birds, Cooper et al.^20^ found no changes in selected vocal muscles of Bengalese finches which exhibited late life declines in temporal and acoustic traits of song (song tempo, pitch, and range of frequency modulation), and suggested instead that such changes may be more likely due to neural deterioration.

Our territory quality index was related to four vocal traits, with our results suggesting that males in higher quality territories produced a higher maximum song frequency, a narrower frequency bandwidth of trills, a shorter trill duration, and a faster trill rate than males in lower quality territories. However, none of these relationships were consistent among models controlling for age, YBD or longevity. Additional analyses (controlling for male age) suggested that territory size was the component of territory quality that was related to maximum song frequency, whereas food availability was related to trill rate and maximum trill frequency. As high quality territories have much denser and taller vegetation than low quality territories^29,52^, it is possible that acoustic transmission may be driving some of these associations. For example, negative relationships between frequency and territory quality may be expected in dense habitats^53,54^, because low frequency sound travels farther through dense vegetation with fewer reverberation effects^54^. However, contrary to this expectation, male Seychelles warblers on high quality territories produced higher maximum song frequencies. Moreover, males on higher quality territories produced faster trill rates, which are not expected to transmit as well through dense vegetation as slower trills^54^. Our results suggest females may be able to obtain better territorial resources by choosing males with certain song characteristics, but more work is needed to determine cause and effect in this species and to disentangle the confounding effects of vegetation density, food availability and male condition on song quality. The relationships involving body mass also varied among models, and should be interpreted cautiously as mass can vary over time and in our study measurement of mass and song recordings were taken 0.6–94 months apart.

In conclusion, our study demonstrates senescence in several acoustic traits of male Seychelles warbler song, but little association between song traits and either future survival or longevity. Our findings highlight the value of long-term studies which document age-dependent changes in song traits during the lifetimes an individuals. Future research should focus on the role of senescent patterns in bird song on age-dependent reproductive success (including extra-pair fertilisations), on proximate explanations of vocal senescence in birds, and on further long-term longitudinal studies of marked populations of species with low extrinsic mortality. The Seychelles warbler would provide an excellent model for such studies, which should provide new insights into the evolutionary pressures driving age-related changes in vocalisations.

## Methods

### Song recordings and field data

Song recordings were made of territory-holding male Seychelles warblers breeding as pairs (i.e. no groups with helpers) on Cousin Island (4°19′53″ S 55°39′47″ E, 29 ha) by J. K. and C. K. Catchpole, using methods described in Catchpole and Komdeur^36^. Recordings were made from 1 to 17 February 1990, which coincides with one of the main breeding periods for this population^55^. Seychelles warblers were individually colour-banded^29^, and the identity of each male was spoken on each recording. At time of recording, Cousin Island supported a population of up to 320 birds on 115–123 breeding territories^37^. The presence of individuals on this island was monitored at least annually from 1981 to 2001^32,40,56^, and for each individual in our dataset the year of hatching and year of presumed death (absence from the island) was recorded^31,40^. From these data we determined: (i) ‘age’ of each male at recording (in years), (ii) ‘longevity’ of each male (years of age at death), and (iii) years from time of recording until death (‘years before death’, YBD). The body mass of males was recorded using a Pesola scale (± 0.1 g). Mass was recorded on the date each individual was caught closest to February 1990 (mean months before or after February 1990 = 32.2 ± 31.3, range 0.6–94.0 months). Territory quality for each male was measured as described in Komdeur (1996), by combining measures of mean annual territory size and monthly food availability measured from 22 January to 12 September 1990. Seychelles warblers are purely insectivorous, taking 98% of their insect food from leaves, so food availability was estimated by multiplying insect abundance on leaves with foliage cover on each territory^29,32,57^.

### Song selection and acoustic analysis

For this study, we defined a song as a series of three or more syllables (element or element groups) (see Supplementary Fig. S1 online). Syllables were separated by an inter-song interval of no larger than 0.6 s, and in Seychelles warblers songs are separated by a clear interval of at least several seconds^36^. Trills were defined as a series of the same syllable repeated three or more times consecutively within the song, with similar inter-trill interval durations between each syllable (see Supplementary Fig. S1 online). Definitions of elements, syllables, and trills (see Supplementary Fig. S1 online), along with inter-song duration, were determined prior to the commencement of data analysis based on subsampling of song recordings of the Seychelles warbler, and methods described in Thompson et al.^58^.

For each male, we created spectrograms of every song and filtered out noise below 700 Hz and above 20,000 Hz. Based on visual assessment of these spectrograms, we excluded any songs which did not contain trills or for which it was not possible to clearly distinguish the song elements from background noise or other background songs. We then selected for acoustic analysis the remaining songs from males with at least one song recording of sufficient quality, up to a maximum of 20 songs per male; where > 20 songs were available 20 songs were selected using a random number generator, and where < 20 songs of sufficient quality were available all songs of sufficient quality for that individual were analysed. This resulted in n = 249 songs for analysis, with 8.0 ± 4.7 songs (range 1–20) per male. We carried out acoustic analysis in Raven Pro 1.5 software, using default settings (Setting: 512 FFT-length, 75% frame, Hamming window, 75% overlap) (Bioacoustics Research Program, Cornell Lab of Ornithology). We then calculated 15 vocal traits for songs and trills, as follows.

First, we measured eleven temporal and frequency variables, which included: (i–ii) duration of trills and songs (seconds), (iii) trill rate (calculated as the number of syllables comprising a trill divided by the duration of the trill, Hz), (iv–v) minimum frequency of trills and songs (Hz), (vi–vii) maximum frequency of trill and songs (Hz), (viii–ix) frequency bandwidth of trills and songs (calculated as the difference between the minimum and maximum frequency, Hz), and (x–xi) peak frequency of songs and trills (Hz). Duration and peak frequency were determined by manually positioning the selection box around trills and songs using a spectrogram in Raven. Bandwidth, minimum and maximum frequency were determined following Zollinger et al.^59^ at − 24 dB below the peak amplitude of the recordings, in order to circumvent the measurement errors that can arise when measuring frequency from spectrograms uncalibrated for amplitude. The threshold of − 24 dB was chosen prior to analysis and matched similar studies^46,60^.

Next, we calculated vocal deviation following the method described in Podos^46^. This method is based on the speed of frequency modulation in a trill, and provides a measure of how challenging a song is to produce because there is expected to be a trade-off between frequency bandwidth and trill rate. In brief, trills were binned into 1-Hz categories (i.e. 1–1.999 Hz, 2–2.999 Hz … 32–32.999 Hz), resulting in 32 bins. For each bin, we regressed mean maximum frequency bandwidth over mean trill rate and took the resulting linear regression line to represent the estimated overall performance limit (linear regression model: trill rate = 28.3 – 0.00953 × frequency bandwidth, r = − 0.460, df = 248). Then, for each song with a sequence of trills, vocal deviation was calculated as the orthogonal distance between the performance limit and the observed vocal performance for each trill. For statistical analysis, we used vocal deviation measured for each trill, and the minimum and mean vocal deviation among all trills recorded for each male, resulting in a further three vocal traits.

Finally, a measure of male repertoire size was generated by adapting methods used by Catchpole and Komdeur^36^, with repertoire size representing the number of unique trill types produced by each male, accounting for the number of songs analysed and the duration of these recordings for each individual. We obtained song recordings from n = 35 males, but four males did not produce trills in any of their recorded songs and were excluded from analyses, resulting in a sample size of n = 31 males (n = 29 for repertoire size).

### Statistics

Statistical analyses were performed in SPSS 21 (IBM Corporation, NY, USA). For the three vocal traits measured at the level of individual males (minimum vocal deviation, mean vocal deviation, and repertoire size), generalised linear models (proc GLM) were used. For remaining vocal traits, linear mixed models (LMM; proc MIXED) were used with a random intercept (Male ID) to account for multiple cases (songs) per male. For each model, male age, YBD, or longevity were included as fixed factors, and tested as both linear and quadratic contrasts to uncover non-linear (senescent) associations. To avoid biased estimates due to collinearity, we present models for each of the fixed factors (age, longevity, or YBD) separately; longevity and YBD were highly correlated (*r* = 0.781), while age was somewhat less strongly correlated with longevity and YBD (*r* = 0.378 and − 0.373, respectively); moreover, models including longevity together with either age or YBD are statistically equivalent^24^. All models also included two other covariates: territory quality, to test for any associations between territory quality and song traits; and body mass, to control for any associations between male size/condition and song traits. Additionally, to gain further insight into the possible roles of the components of territory quality in song variation, we then repeated models but with the covariate territory quality replaced by the two variables from which it was calculated: (i) territory size, and (ii) availability of insect food (foliage cover x insect density), whilst controlling for age and body mass. We also checked for interactions between territory quality and age, YBD or longevity whilst controlling for body mass (only significant interactions reported). We analysed song variables using separate models, rather than employing a dimension reduction technique such as principal component analysis, to allow clearer interpretability and gain insight into the relationships between discrete acoustic traits and age. While we acknowledge that care is required when interpreting results of multiple comparisons, we did not apply a correction of the family-wise error rate as our contrasts were planned, we were studying a complex response, and prevailing methods for such corrections are overly conservative and may lead to unacceptably high type II error rates, particularly when analysing correlated responses as is often the case in multivariate ecological studies such as this^61–63^. However, to counter this problem we present in the results, in addition to individual tests and estimates of effect size, an overall test of the linear and quadratic relationships of age, YBD and longevity, by counting the number of significant results for each predictor and using a Bernoulli process to calculate the probability of finding that number by chance alone^61^. Means are reported ± standard deviation.

## Supplementary information

Supplementary Information.

## Data Availability

The datasets analysed during the current study are available from the corresponding author on reasonable request.
