# Author Correction: Senescence of song revealed by a long-term study of the Seychelles warbler (*Acrocephalus sechellensis*)

**DOI:** 10.1038/s41598-021-88215-6

**Published:** 2021-04-15

**Authors:** Mathew L. Berg, Sarah C. Beebe, Jan Komdeur, Adam P. A. Cardilini, Raoul F. H. Ribot, Andrew T. D. Bennett, Katherine L. Buchanan

**Affiliations:** 1 Centre for Integrative Ecology, School of Life and Environmental Sciences, Deakin University, Locked Bag 20000, Geelong, VIC 3220 Australia; 2 Groningen Institute for Evolutionary Life Sciences, Faculty of Science and Engineering, University of Groningen, Nijenborgh 7, 9747 AG Groningen, The Netherlands; 3 Department of Zoology, University of Cambridge, Downing Street, Cambridge, CB2 4EJ UK; 4 Nature Seychelles, Victoria, Mahé Seychelles

Correction to: *Scientific Reports* 10.1038/s41598-020-77405-3, published online 24 November 2020

The original version of this Article contained an error. An affiliation was omitted for the author Jan Komdeur, which is now listed as Affiliation 4:

Nature Seychelles, Victoria, Mahé, Seychelles

This has now been corrected in the HTML and PDF versions of this Article, and in the accompanying Supplemental Material.

