# Supplementary material for: Senescence of song revealed by a long-term study of the Seychelles warbler (Acrocephalus sechellensis)
Source: Sci Rep. 2020 Nov 24;10:20479. doi: 10.1038/s41598-020-77405-3 (PMC7686343; doi:10.1038/s41598-020-77405-3)
Supplement: Supplementary file 1 — Supplementary Information. [file 41598_2020_77405_MOESM1_ESM.docx]

**Supplementary Material**

**SENESCENCE OF SONG REVEALED BY A LONG-TERM STUDY OF THE SEYCHELLES WARBLER (*ACROCEPHALUS SECHELLENSIS*)**

Mathew L. Berg^1^*, Sarah C. Beebe^1^, Jan Komdeur^2,3,4^, Adam P. A. Cardilini^1^, Raoul F. H. Ribot^1^, Andrew T. D. Bennett^1^, and Katherine L. Buchanan^1^

^1^ School of Life and Environmental Sciences, Centre for Integrative Ecology, Deakin University, Locked Bag 20000, Geelong, Victoria 3220, Australia

^2^ Groningen Institute for Evolutionary Life Sciences, Faculty of Science and Engineering, University of Groningen, Nijenborgh 7, 9747 AG, The Netherlands

^3^ Department of Zoology, University of Cambridge, Downing Street, Cambridge, CB2 4EJ, UK

^4^ Nature Seychelles, Victoria, Mahé, Seychelles

**Supplementary Figure S1a**

**Supplementary Figure S1b**

**
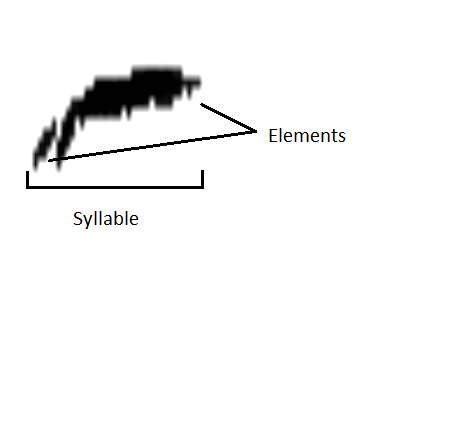
**

**Supplementary Figure S1.** (a) Spectrogram (frequency, kHz, plotted against time, s) of a typical song produced by a territory owning male from the Cousin Island population of the Seychelles warbler in February 1990, showing the length of song elements and frequency characteristics used in this study. (b) An example of a syllable constructed from two unique elements.

| **Supplementary Table S1.** Associations of years before death at time of recording (YBD; showing linear and quadratic contrasts), territory quality and body mass with male song traits in Seychelles warblers | | | | | | |
| --- | --- | --- | --- | --- | --- | --- |
| Song trait | Predictors | Estimate | SE | *t* | df | *P* |
| Trill duration (s) | YBD (linear) | -1.25 | 2.54 | -0.49 | 2.42 | 0.663 |
|  | YBD (quadratic) | -2.27 | 2.32 | -0.98 | 2.30 | 0.420 |
|  | Territory quality | -0.11 | 0.05 | -2.36 | 3.14 | 0.096 |
|  | Mass | -0.06 | 0.06 | -1.02 | 2.46 | 0.399 |
| Song duration (s) | YBD (linear) | -1.51 | 2.78 | -0.54 | 1.39 | 0.661 |
|  | YBD (quadratic) | -3.94 | 2.60 | -1.52 | 1.43 | 0.314 |
|  | Territory quality | -0.08 | 0.05 | -1.56 | 2.36 | 0.241 |
|  | Mass | -0.15 | 0.07 | -2.20 | 1.44 | 0.206 |
| **Trill rate (Hz)** | YBD (linear) | -6.81 | 17.03 | -0.40 | 113.00 | 0.690 |
|  | YBD (quadratic) | 5.90 | 15.32 | 0.39 | 113.00 | 0.701 |
|  | **Territory quality** | 1.75 | 0.32 | 5.43 | 113.00 | **<0.001** |
|  | **Mass** | 1.13 | 0.42 | 2.71 | 113.00 | **0.008** |
| Min. song frequency (Hz) | YBD (linear) | -199.67 | 1669.71 | -0.12 | 4.65 | 0.91 |
|  | YBD (quadratic) | -1193.11 | 1551.69 | -0.77 | 4.52 | 0.48 |
|  | Territory quality | 8.13 | 28.85 | 0.28 | 5.21 | 0.789 |
|  | Mass | 2.97 | 41.31 | 0.07 | 4.40 | 0.946 |
| Max. song frequency (Hz) | YBD (linear) | 94.42 | 2658.79 | 0.04 | 2.45 | 0.974 |
|  | YBD (quadratic) | -4688.30 | 2409.02 | -1.95 | 2.12 | 0.184 |
|  | Territory quality | -3.40 | 49.31 | -0.07 | 3.34 | 0.949 |
|  | Mass | -89.90 | 61.67 | -1.46 | 1.65 | 0.306 |
| Song bandwidth (Hz) | YBD (linear) | 88.09 | 1999.47 | 0.04 | 1.96 | 0.969 |
|  | YBD (quadratic) | -5538.18 | 1866.26 | -2.97 | 2.01 | 0.097 |
|  | Territory quality | -12.40 | 37.67 | -0.33 | 3.22 | 0.762 |
|  | Mass | -101.66 | 50.25 | -2.02 | 2.04 | 0.178 |
| Min. trill frequency (Hz) | YBD (linear) | -516.32 | 3376.51 | -0.15 | 4.91 | 0.885 |
|  | YBD (quadratic) | -1470.33 | 3115.91 | -0.47 | 4.83 | 0.658 |
|  | Territory quality | 59.76 | 57.14 | 1.05 | 5.54 | 0.339 |
|  | Mass | 67.55 | 82.86 | 0.82 | 5.10 | 0.451 |
| Max. trill frequency (Hz) | YBD (linear) | -668.48 | 4040.81 | -0.17 | 3.71 | 0.877 |
|  | YBD (quadratic) | -4666.49 | 3724.60 | -1.25 | 3.63 | 0.285 |
|  | Territory quality | 5.43 | 68.93 | 0.08 | 4.31 | 0.941 |
|  | Mass | -22.43 | 99.39 | -0.23 | 3.86 | 0.833 |
| **Trill bandwidth (Hz)** | YBD (linear) | 221.48 | 1699.77 | 0.13 | 113.00 | 0.897 |
|  | **YBD (quadratic)** | -3647.07 | 1529.31 | -2.39 | 113.00 | **0.019** |
|  | **Territory quality** | -65.73 | 32.09 | -2.05 | 113.00 | **0.043** |
|  | **Mass** | -101.59 | 41.65 | -2.44 | 113.00 | **0.016** |
| Peak trill frequency (Hz) | YBD (linear) | -1341.01 | 3902.61 | -0.34 | 4.52 | 0.747 |
|  | YBD (quadratic) | -6258.51 | 3604.27 | -1.74 | 4.46 | 0.15 |
|  | Territory quality | -48.09 | 65.67 | -0.73 | 4.99 | 0.497 |
|  | Mass | -105.75 | 95.58 | -1.11 | 4.68 | 0.322 |
| Peak song frequency (Hz) | YBD (linear) | -2102.06 | 4089.66 | -0.51 | 4.25 | 0.633 |
|  | YBD (quadratic) | -5023.54 | 3797.18 | -1.32 | 4.28 | 0.252 |
|  | Territory quality | -63.63 | 69.74 | -0.91 | 4.95 | 0.404 |
|  | Mass | -88.34 | 100.66 | -0.88 | 4.48 | 0.425 |
| Vocal deviation | YBD (linear) | 3.76 | 20.71 | 0.18 | 3.97 | 0.865 |
|  | YBD (quadratic) | 29.37 | 18.64 | 1.58 | 3.48 | 0.201 |
|  | Territory quality | -0.56 | 0.39 | -1.44 | 6.02 | 0.199 |
|  | Mass | 0.68 | 0.51 | 1.35 | 3.45 | 0.259 |
| Minimum vocal deviation | YBD (linear) |  | 33.77 | 0.20 | 6.00 | 0.195 |
|  | YBD (quadratic) | 22.88 | 31.29 | 0.73 | 6.00 | 0.492 |
|  | Territory quality | -0.24 | 0.55 | -0.44 | 6.00 | 0.678 |
|  | Mass | -0.69 | 0.82 | -0.84 | 6.00 | 0.434 |
| Mean vocal deviation | YBD (linear) | 3.49 | 21.41 | 0.16 | 6.00 | 0.876 |
|  | YBD (quadratic) | 34.67 | 19.84 | 1.75 | 6.00 | 0.131 |
|  | Territory quality | -0.49 | 0.35 | -1.41 | 6.00 | 0.209 |
|  | Mass | 0.49 | 0.52 | 0.94 | 6.00 | 0.385 |
| Repertoire size | YBD (linear) | -0.71 | 1.15 | -0.62 | 6.00 | 0.558 |
|  | YBD (quadratic) | 0.11 | 1.06 | 0.11 | 6.00 | 0.919 |
|  | Territory quality | 0.02 | 0.02 | 0.87 | 6.00 | 0.417 |
|  | Mass | 0.00 | 0.03 | 0.11 | 6.00 | 0.920 |
|  |  |  |  |  |  |  |
| Significant predictors (P < 0.05) are highlighted in bold. | | | |  |  |  |

| **Supplementary Table S2.** Associations of longevity (showing linear and quadratic contrasts), territory quality and body mass with male song traits in Seychelles warblers | | | | | | |
| --- | --- | --- | --- | --- | --- | --- |
| Song trait | Predictors | Estimate | SE | *t* | df | *P* |
| **Trill duration (s)** | Longevity (linear) | -2.05 | 2.95 | -0.70 | 111.00 | 0.488 |
|  | Longevity (quadratic) | -3.24 | 1.95 | -1.66 | 111.00 | 0.100 |
|  | **Territory quality** | -0.13 | 0.05 | -2.41 | 111.00 | **0.018** |
|  | **Mass** | -0.24 | 0.09 | -2.56 | 111.00 | **0.012** |
| Song duration (s) | Longevity (linear) | -4.51 | 6.27 | -0.72 | 4.44 | 0.508 |
|  | Longevity (quadratic) | -6.06 | 4.32 | -1.41 | 4.07 | 0.231 |
|  | Territory quality | -0.27 | 0.11 | -2.37 | 4.55 | 0.069 |
|  | Mass | -0.23 | 0.18 | -1.26 | 6.68 | 0.250 |
| **Trill rate (Hz)** | Longevity (linear) | -43.23 | 31.47 | -1.37 | 111.00 | 0.172 |
|  | Longevity (quadratic) | -24.64 | 20.80 | -1.19 | 111.00 | 0.239 |
|  | Territory quality | 0.37 | 0.57 | 0.66 | 111.00 | 0.514 |
|  | **Mass** | 3.07 | 0.99 | 3.11 | 111.00 | **0.002** |
| Min. song frequency (Hz) | Longevity (linear) | -743.40 | 526.90 | -1.41 | 4.11 | 0.229 |
|  | Longevity (quadratic) | -9318.14 | 4503.76 | -2.07 | 3.63 | 0.115 |
|  | Territory quality | -10.98 | 21.98 | -0.50 | 3.44 | 0.647 |
|  | Mass | -4.10 | 36.37 | -0.11 | 5.65 | 0.914 |
| Max. song frequency (Hz) | Longevity (linear) | -343.50 | 1822.86 | -0.19 | 4.45 | 0.859 |
|  | Longevity (quadratic) | -7898.54 | 15643.80 | -0.51 | 3.98 | 0.640 |
|  | Territory quality | -42.34 | 76.46 | -0.55 | 3.80 | 0.611 |
|  | Mass | -200.34 | 124.63 | -1.61 | 5.99 | 0.159 |
| Song bandwidth (Hz) | Longevity (linear) | 2041.74 | 4906.19 | 0.42 | 4.29 | 0.687 |
|  | Longevity (quadratic) | -1640.92 | 3393.87 | -0.48 | 4.01 | 0.654 |
|  | Territory quality | -4.93 | 89.40 | -0.06 | 4.40 | 0.958 |
|  | Mass | -157.42 | 140.26 | -1.12 | 6.10 | 0.304 |
| Min. trill frequency (Hz) | Longevity (linear) | -6072.48 | 4696.84 | -1.29 | 4.25 | 0.262 |
|  | Longevity (quadratic) | -3139.60 | 3246.65 | -0.97 | 3.96 | 0.389 |
|  | Territory quality | 52.86 | 85.15 | 0.62 | 4.29 | 0.566 |
|  | Mass | 60.87 | 131.26 | 0.46 | 5.62 | 0.660 |
| Max. trill frequency (Hz) | Longevity (linear) | -4958.35 | 5148.76 | -0.96 | 4.52 | 0.384 |
|  | Longevity (quadratic) | -4417.76 | 3526.61 | -1.25 | 4.07 | 0.278 |
|  | Territory quality | 15.55 | 93.43 | 0.17 | 4.56 | 0.875 |
|  | Mass | -175.29 | 148.40 | -1.18 | 6.53 | 0.279 |
| Trill bandwidth (Hz) | Longevity (linear) | 2950.45 | 3811.28 | 0.77 | 5.93 | 0.469 |
|  | Longevity (quadratic) | -1023.55 | 2571.04 | -0.40 | 5.05 | 0.707 |
|  | Territory quality | -49.23 | 69.23 | -0.71 | 5.98 | 0.504 |
|  | Mass | -219.75 | 114.60 | -1.92 | 9.35 | 0.086 |
| Peak trill frequency (Hz) | Longevity (linear) | 1968.52 | 5146.53 | 0.38 | 4.22 | 0.721 |
|  | Longevity (quadratic) | -1155.97 | 3564.33 | -0.32 | 3.96 | 0.762 |
|  | Territory quality | -7.87 | 93.29 | -0.08 | 4.25 | 0.937 |
|  | Mass | -133.22 | 142.82 | -0.93 | 5.45 | 0.390 |
| Peak song frequency (Hz) | Longevity (linear) | 711.35 | 5851.22 | 0.12 | 4.06 | 0.909 |
|  | Longevity (quadratic) | 70.05 | 4069.25 | 0.02 | 3.87 | 0.987 |
|  | Territory quality | -22.65 | 106.41 | -0.21 | 4.14 | 0.842 |
|  | Mass | -111.58 | 162.87 | -0.69 | 5.31 | 0.522 |
| Vocal deviation | Longevity (linear) | -31.18 | 60.48 | -0.52 | 4.58 | 0.630 |
|  | Longevity (quadratic) | 13.62 | 41.52 | 0.33 | 4.16 | 0.759 |
|  | Territory quality | -0.39 | 1.10 | -0.36 | 4.62 | 0.738 |
|  | Mass | 0.03 | 1.73 | 0.02 | 6.48 | 0.988 |
| Minimum vocal deviation | Longevity (linear) | 28.64 | 51.06 | 0.56 | 4.00 | 0.605 |
|  | Longevity (quadratic) | -25.49 | 35.94 | -0.71 | 4.00 | 0.517 |
|  | Territory quality | -0.76 | 0.92 | -0.83 | 4.00 | 0.456 |
|  | Mass | -0.52 | 1.32 | -0.40 | 4.00 | 0.712 |
| Mean vocal deviation | Longevity (linear) | -34.62 | 57.78 | -0.60 | 4.00 | 0.581 |
|  | Longevity (quadratic) | 12.06 | 40.68 | 0.30 | 4.00 | 0.782 |
|  | Territory quality | -0.45 | 1.05 | -0.43 | 4.00 | 0.692 |
|  | Mass | 0.16 | 1.49 | 0.10 | 4.00 | 0.922 |
| Repertoire size | Longevity (linear) | -2.62 | 1.63 | -1.61 | 4.00 | 0.182 |
|  | Longevity (quadratic) | -1.35 | 1.14 | -1.18 | 4.00 | 0.303 |
|  | Territory quality | -0.06 | 0.03 | -1.99 | 4.00 | 0.118 |
|  | Mass | 0.03 | 0.04 | 0.66 | 4.00 | 0.544 |
|  |  |  |  |  |  |  |
| Significant predictors (P < 0.05) are highlighted in bold. | | |  |  |  |  |

| **Supplementary Table S3.** Associations of each quantified component of territory quality (territory size, vegetation density x insect density per unit vegetation) with song traits in male Seychelles warblers, controlling for age and body mass | | | | | | |
| --- | --- | --- | --- | --- | --- | --- |
| Song trait | Predictors | Estimate | SE | *t* | df | *P* |
| Trill duration (s) | Age (linear) | -4.62 | 2.19 | -2.12 | 5.02 | 0.088 |
|  | **Age (quadratic)** | -7.07 | 2.64 | -2.68 | 13.69 | **0.018** |
|  | **Mass** | -0.18 | 0.07 | -2.71 | 4.35 | **0.049** |
|  | Territory size | 0.07 | 0.73 | 0.10 | 3.16 | 0.926 |
|  | Vegetation+insects | -0.02 | 0.01 | -1.67 | 17.57 | 0.113 |
| Song duration (s) | Age (linear) | -8.82 | 4.17 | -2.12 | 5.16 | 0.086 |
|  | **Age (quadratic)** | -12.22 | 4.58 | -2.67 | 9.98 | **0.024** |
|  | Mass | -0.32 | 0.13 | -2.48 | 5.03 | 0.056 |
|  | Territory size | 0.87 | 1.47 | 0.59 | 4.13 | 0.588 |
|  | Vegetation+insects | -0.01 | 0.02 | -0.78 | 12.18 | 0.451 |
| Trill rate (Hz) | Age (linear) | -7.54 | 19.04 | -0.40 | 123.00 | 0.693 |
|  | Age (quadratic) | 15.54 | 23.93 | 0.65 | 123.00 | 0.517 |
|  | **Mass** | 1.17 | 0.58 | 2.02 | 123.00 | **0.046** |
|  | Territory size | 9.62 | 6.16 | 1.56 | 123.00 | 0.121 |
|  | **Vegetation+insects** | 0.26 | 0.09 | 2.90 | 123.00 | **0.004** |
| Min. song frequency (Hz) | Age (linear) | 549.72 | 698.30 | 0.79 | 89.00 | 0.433 |
|  | Age (quadratic) | 511.22 | 993.78 | 0.51 | 89.00 | 0.608 |
|  | **Mass** | 60.99 | 24.21 | 2.52 | 89.00 | **0.014** |
|  | **Territory size** | 913.42 | 247.20 | 3.70 | 89.00 | **<0.001** |
|  | Vegetation+insects | -5.81 | 3.61 | -1.61 | 89.00 | 0.111 |
| Max. song frequency (Hz) | Age (linear) | -3043.13 | 2235.43 | -1.76 | 89.00 | 0.081 |
|  | Age (quadratic) | -4371.62 | 3181.32 | -1.37 | 89.00 | 0.173 |
|  | Mass | -124.31 | 77.50 | -1.60 | 89.00 | 0.112 |
|  | **Territory size** | 2014.57 | 791.34 | 2.55 | 89.00 | **0.013** |
|  | Vegetation+insects | -7.77 | 11.56 | -0.67 | 89.00 | 0.503 |
| Song bandwidth (Hz) | Age (linear) | -3288.27 | 2899.43 | -1.13 | 3.66 | 0.326 |
|  | Age (quadratic) | -5553.90 | 3109.17 | -1.79 | 6.47 | 0.121 |
|  | Mass | -113.88 | 90.43 | -1.26 | 3.64 | 0.283 |
|  | Territory size | 1561.86 | 1035.89 | 1.51 | 3.05 | 0.227 |
|  | Vegetation+insects | -2.73 | 11.35 | -0.24 | 7.73 | 0.816 |
| Min. trill frequency (Hz) | Age (linear) | 650.75 | 2808.59 | 0.23 | 5.58 | 0.825 |
|  | Age (quadratic) | 2285.56 | 2931.97 | 0.78 | 8.75 | 0.456 |
|  | Mass | 151.76 | 87.34 | 1.74 | 5.63 | 0.136 |
|  | Territory size | 1629.20 | 1006.52 | 1.62 | 4.75 | 0.169 |
|  | Vegetation+insects | -10.21 | 10.58 | -0.97 | 9.86 | 0.357 |
| Max. trill frequency (Hz) | Age (linear) | -864.58 | 3072.86 | -0.28 | 4.30 | 0.791 |
|  | Age (quadratic) | -1648.91 | 3355.97 | -0.49 | 8.08 | 0.636 |
|  | Mass | 90.02 | 95.53 | 0.94 | 4.22 | 0.397 |
|  | Territory size | 2839.79 | 1079.52 | 2.63 | 3.38 | 0.069 |
|  | **Vegetation+insects** | -29.00 | 12.23 | -2.37 | 9.55 | **0.040** |
| Trill bandwidth (Hz) | Age (linear) | -2482.92 | 1930.32 | -1.29 | 123.00 | 0.201 |
|  | **Age (quadratic)** | -5608.31 | 2425.60 | -2.31 | 123.00 | **0.022** |
|  | Mass | -116.62 | 58.85 | -1.98 | 123.00 | 0.050 |
|  | Territory size | 792.68 | 624.00 | 1.27 | 123.00 | 0.206 |
|  | Vegetation+insects | -13.32 | 9.08 | -1.47 | 123.00 | 0.145 |
| Peak trill frequency (Hz) | Age (linear) | -1181.66 | 3369.57 | -0.35 | 5.01 | 0.740 |
|  | Age (quadratic) | -5816.94 | 3423.20 | -1.70 | 7.09 | 0.133 |
|  | Mass | -94.97 | 104.69 | -0.91 | 5.09 | 0.405 |
|  | Territory size | 1405.21 | 1220.68 | 1.15 | 4.45 | 0.308 |
|  | Vegetation+insects | -8.99 | 12.27 | -0.73 | 7.77 | 0.485 |
| Peak song frequency (Hz) | Age (linear) | -3398.98 | 2995.16 | -1.14 | 4.01 | 0.320 |
|  | Age (quadratic) | -6992.24 | 3176.36 | -2.20 | 6.78 | 0.065 |
|  | Mass | -135.05 | 93.41 | -1.45 | 4.03 | 0.221 |
|  | Territory size | 461.34 | 1074.53 | 0.43 | 3.41 | 0.693 |
|  | Vegetation+insects | -0.75 | 11.56 | -0.07 | 7.98 | 0.950 |
| Vocal deviation | Age (linear) | 21.03 | 26.88 | 0.78 | 4.53 | 0.473 |
|  | Age (quadratic) | 51.40 | 31.72 | 1.62 | 11.44 | 0.132 |
|  | Mass | 0.41 | 0.83 | 0.49 | 4.07 | 0.651 |
|  | Territory size | -9.74 | 9.07 | -1.07 | 3.02 | 0.361 |
|  | Vegetation+insects | -0.10 | 0.12 | -0.84 | 14.48 | 0.417 |
| Min. vocal deviation | Age (linear) | 34.19 | 46.79 | 0.93 | 6.00 | 0.389 |
|  | Age (quadratic) | 46.68 | 34.20 | 1.37 | 6.00 | 0.221 |
|  | Mass | -0.30 | 1.13 | -0.26 | 6.00 | 0.801 |
|  | Territory size | -3.37 | 13.73 | -0.25 | 6.00 | 0.814 |
|  | Vegetation+insects | 0.00 | 0.12 | -0.01 | 6.00 | 0.995 |
| Mean vocal deviation | Age (linear) | 10.93 | 27.60 | 0.40 | 6.00 | 0.706 |
|  | Age (quadratic) | 45.19 | 25.66 | 1.76 | 6.00 | 0.129 |
|  | Mass | -0.11 | 0.85 | -0.13 | 6.00 | 0.900 |
|  | Territory size | -13.28 | 10.30 | -1.29 | 6.00 | 0.245 |
|  | Vegetation+insects | -0.10 | 0.09 | -1.11 | 6.00 | 0.310 |
| Repertoire size | Age (linear) | -0.87 | 1.38 | -0.64 | 6.00 | 0.548 |
|  | Age (quadratic) | -0.92 | 1.28 | -0.72 | 6.00 | 0.501 |
|  | Mass | 0.01 | 0.04 | 0.17 | 6.00 | 0.871 |
|  | Territory size | 0.52 | 0.51 | 1.02 | 6.00 | 0.348 |
|  | Vegetation+insects | 0.00 | 0.00 | -0.45 | 6.00 | 0.668 |
|  |  |  |  |  |  |  |
| Significant predictors (P < 0.05) are highlighted in bold. | | | |  |  |  |
